# Does gadolinium truly enhance the active surveillance of men with prostate cancer?

**DOI:** 10.1002/bco2.70296

**Published:** 2026-10-09

**Authors:** Abinav Udaiyar, Navid Jabalameli, Soroush Rais‐Bahrami

**Affiliations:** ^1^ Department of Urology Wake Forest University School of Medicine Winston‐Salem North Carolina USA; ^2^ Department of Radiology Wake Forest University School of Medicine Winston‐Salem North Carolina USA; ^3^ Atrium Health Wake Forest Baptist Comprehensive Cancer Center Wake Forest University School of Medicine Winston‐Salem North Carolina USA; ^4^ Department of Cancer Medicine Wake Forest University School of Medicine Winston‐Salem North Carolina USA

**Keywords:** active surveillance, biparametric MRI, gadolinium, multiparametric MRI, PI‐RADS, prostate cancer

Active surveillance (AS) is the preferred management of low‐risk prostate cancer (PCa) and has expanded to some favourable intermediate‐risk cases, and multiparametric MRI (mpMRI) plays a significant role in monitoring patients who are on surveillance. However, widespread adoption of serial mpMRI is challenging because of resource constraints and increased demand for prostate MRI. Biparametric MRI (bpMRI) omits the gadolinium‐based dynamic contrast‐enhanced (DCE) phase, retaining only the T2‐weighted and diffusion‐weighted sequences that carry most of the diagnostic signal.

In this setting, we eagerly read the paper by Rivera López and colleagues, which asked a simple but impactful question: Can the DCE sequence be safely dropped from surveillance MRI?^1^ Most published bpMRI versus mpMRI comparisons have been small, single‐centre, unblinded and retrospective, without MRI quality assurance^2^; this study represents single‐centre and retrospective data as well but brings a mature AS database, biopsy‐verified endpoints and a matched control cohort. Among 129 men on AS, and a 68‐man biopsied subgroup matched to 68 mpMRI controls, bpMRI performed comparably to mpMRI: Grade reclassification occurred in 29% vs. 25% (*p* = 0.7); extreme reclassification to GG ≥ 3 was identical at 7.3%, and no difference was found in PI‐RADS lesion stability. This is a timely, pragmatic contribution since bpMRI in the biopsy‐naïve setting is now supported by Level 1 evidence from the prospective, multicentre PRIME trial, with early evidence of its benefit developing for over a decade.^2^, ^3^ However, the role of bpMRI during AS, where imaging is repeated sometimes several times over consecutive years, still lacks robust data.

Within PI‐RADS v2.1, DCE only contributes to the scoring by upgrading an equivocal peripheral zone diffusion finding from PI‐RADS 3 to PI‐RADS 4.^4^ In AS programmes that biopsy every PI‐RADS ≥3 lesion, this does not change clinical practice since the lesions found are biopsied in either scenario. The study is built on this rationale: Every patient had a prior mpMRI localizing known disease, and all PI‐RADS 3–5 lesions are targeted at the time of surveillance biopsy, so the incremental yield of gadolinium use for mpMRI in their AS programme should be minimal.

The stakes go beyond convenience. Men on AS accumulate serial contrast exposures, and trace gadolinium is retained with all agents, although no adverse clinical effect has been demonstrated. The case for omission rests less on toxicity, which remains unsettled, than on proportionality: An agent that alters no management decision should not be given repeatedly across potentially a decade of surveillance in a low‐risk patient population. Dropping the contrast also shortens scans, removes intravenous access, improves throughput and lowers costs, which may widen access to surveillance imaging.^5^

The study's principal limitation is one of design. Each man contributes an mpMRI, and a median of 29 months later, a bpMRI, so the modality is confounded by more than 2 years of disease evolution. The sample is modest, with a 4% reclassification gap, which fails to detect a difference rather than proving equivalence. Additionally, the bpMRI arm's excess of Grade Group 2 disease (9 vs. 2) runs against the feared bias, strengthening rather than weakening the comparison. Every patient had a prior mpMRI, and the authors rightly declined to endorse bpMRI as the sole surveillance modality based upon this aspect of their study design. The greater downgrading of PI‐RADS 4/5 lesions to PI‐RADS 3 in the bpMRI group (6 of 26 vs. 1 of 28) is the expected footprint of withdrawing DCE, and a caution: such a lesion has not regressed, so a programme that uses PI‐RADS change to trigger biopsy would mistake a protocol artefact for improvement. The two protocols are interchangeable not at the level of the individual score but at the level of a programme that biopsies every PI‐RADS ≥3 lesion.

The recurring objection to dropping DCE is that it removes a safety net when diffusion imaging is degraded and that performance may falter in less‐experienced hands outside high‐volume, expert centres. However, one potential remedy that may become available soon is artificial intelligence (AI), and it may address both. Deep‐learning decision support in radiology is well underway and has been trained on T2‐weighted and diffusion data, making it biparametric. In a 61‐reader international study, AI assisted non‐expert radiologists reach expert‐level accuracy and improves interreader agreement on equivocal PI‐RADS 3 lesions where DCE was once the mediator.^6^ That evidence is from the diagnostic setting; however, the future of AS may well be contrast‐free acquisition paired with AI‐assisted reading that could be made reproducible beyond tertiary referral centres.

This study supports bpMRI for men stably on AS who already have an mpMRI, in programmes that biopsy every PI‐RADS ≥3 lesion: not the diagnostic scan and not a programme that watches PI‐RADS 3 lesions rather than sampling them. Settling the remainder needs a prospective surveillance cohort with paired same‐visit reads and lesion‐level scoring on a protocol that does not switch modality midway. Within those limits, gadolinium enhances the image, not the surveillance.

## FUNDING INFORMATION

There was no funding for this work.

## CONFLICT OF INTEREST STATEMENT

Abinav Udaiyar and Navid Jabalemli have no disclosures. Soroush Rais‐Bahrami serves as a consultant to Artera AI, Blue Earth Diagnostics, Ferring Pharmaceuticals, FocalTherics, Intuitive Surgical, Philips Corp and Progenics.

## Data Availability

Data sharing is not applicable to this article as no datasets were generated or analysed during the current study.
